# Intraspecific diversity of durum wheat (Triticum durum Desf.): a unif ied classif ication

**DOI:** 10.18699/VJ21.029

**Published:** 2021-05

**Authors:** O.A. Lyapunova

**Affiliations:** Federal Research Center the N.I. Vavilov All-Russian Institute of Plant Genetic Resources (VIR), St. Petersburg, Russia

**Keywords:** durum wheat (Triticum durum), intraspecific classification, complexes of morphological traits, inheritance of traits, botanical variety, твердая пшеница (Triticum durum), внутривидовая классификация, комплексы морфологических признаков, наследование признаков, ботаническая разновидность

## Abstract

The Department of Wheat Genetic Resources of the All-Russian Research Institute of Plant Genetic Resources (VIR) had developed and published in 1979 a classification of the genus Triticum L., which is based on the genomic composition of species and the presence or absence of a number of main genes that govern the “classification” traits. The grounds have been laid by F. Körnicke and J. Percival, and supplemented by N.I. Vavilov and K.A. Flaksberger. The classification, which is most often referred to as the “Classification of Triticum by Dorofeev et al.”, belongs to a number of the main modern classifications of the genus. This is the world’s first standardized system that contains all known intraspecific (infraspecific) taxa of wild and cultivated wheat species. A detailed classification makes it possible to identify a wide variety of forms in the genus Triticum L. and its individual species, which is especially important for collections preserved in genetic seed banks. The use of the intraspecific classification of the genus Triticum L. greatly simplifies the identification of the VIR collection accessions introduced from various sources or checking accession identity after regeneration in the field. However, the direct use of such a voluminous classification meets several difficulties. Therefore, we propose a unified intraspecific classification of durum wheat, based on the description of only 16 main botanical varieties out of 131 described so far, which have complexes of morphological traits of the spike and kernel that occur most frequently in durum wheat collections. The remaining 115 botanical varieties, which have additional traits, get their name by the addition of the abbreviated Latin name of one or another additional trait to the main name. Having mastered this way of describing the morphological traits of accessions, any user can easily navigate oneself in the systematized intraspecific diversity of collections. The purpose of this work is to acquaint the reader with the intraspecific classification of durum wheat (Triticum durum Desf.) developed at VIR and to offer its simplified version, which is based on the identification of the main and additional morphological traits of the spike and kernel.

## Abstract (ru)

В отделе генетических ресурсов пшеницы ВНИИ генетических ресурсов растений (ВИР) разработана и в 1979 г. опубликована система рода Triticum L., базирующаяся на учете геномного состава видов и наличии или отсутствии у них ряда главных генов, контролирующих «классификационные» признаки. Она основана на исследованиях F. Körnicke и J. Percival, дополненных Н.И. Вавиловым и К.А. Фляксбергером. Эту систему, известную как “Classification of Triticum by Dorofeev et al.”, относят к ряду основных современных классификаций рода. Это первая в мире стандартизированная система, содержащая все известные внутривидовые (инфраспецифические) таксоны диких и культурных видов пшеницы. Она дает возможность идентификации большого разнообразия форм при работе с родом Triticum L. и отдельными его видами, что особенно важно для коллекций, сохраняемых в генетических банках семян. Применение внутривидовой классификации рода Triticum L. для идентификации образцов коллекции ВИР, интродуцированных из различных источников или поступивших после полевого размножения для пополнения репродукции образцов, значительно упрощает этот процесс. Однако прямое использование такой объемной классификации связано с рядом трудностей. Поэтому нами предлагается унифицированная внутривидовая классификация твердой пшеницы лишь 16 из 131 разновидности, описанной к настоящему времени, которые обладают наиболее часто встречающимися в коллекциях твердой пшеницы комплексами морфологических признаков колоса и зерновки. Остальные 115 разновидностей, имеющих дополнительные признаки, получают свое название путем добавления к основному названию сокращенного латинского названия того или иного дополнительного признака. Владение таким способом описания морфологических признаков образцов может помочь любому пользователю легко ориентироваться в систематизированном внутривидовом разнообразии коллекций. Цель данной статьи – познакомить читателя с внутривидовой классификацией твердой пшеницы (Triticum durum Desf.), разработанной в ВИР, и предложить ее упрощенный вариант, построенный на выделении главных и дополнительных морфологических признаков колоса и зерновки.

## Introduction

Durum wheat (Triticum durum Desf.) is characterized by a wide diversity of varieties and forms. Like any set, this diversity should be systematized to better understand the relationships between its constituent units. Classification (from the Latin word classis – category, class, and facio – do, make) is a method aimed at organizing a system of subordinate groups, in which these units are combined on the basis of similarity in certain essential properties (Subbotin, 2001). The product of the classification is a system. Plant systematics is a branch of botany that deals with the classification of plants. The term “systematic” (systematic botany) was introduced by the Swedish naturalist Carl von Linné in 1751 in his work “Philosophy of Botany” (Linnaeus, 1989). The term “taxonomy” was introduced by the Swiss botanist Augustin Pyrame de Candolle, the creator of the natural system of plants classification – the de Candolle system – and designated the theory of plant classification, according to the rules of which taxa are arranged in the system (de Candolle, 1813). In his treatise “On the Origin of Species…”, the English naturalist Charles Robert Darwin considered the terms taxonomy and systematic as synonyms (Darwin, 1859). However, systematics studies not only the diversity of organisms, but also the causes and ways of its appearance, and includes taxonomy and nomenclature.

The history of the genus Triticum L. classification begins with C. Linnaeus (Linnaeus, 1737), who is considered by most triticologists as the author of the genus Wheat. Over 300 years of its existence, the Linnaeus classification has undergone numerous interpretations, which are associated with the inclusion or subsequent exclusion of certain cultivated and wild species from it.

The system of the genus Triticum L. developed at the Department of Wheat Genetic Resources of Federal Research Center the N.I. Vavilov All-Russian Institute of Plant Genetic Resources (VIR) (Dorofeev et al., 1979), was built up on the research of such triticologists as F. Körnicke (1885) and J. Percival (1921), and further revised and supplemented by N.I. Vavilov (1935) and K.A. Flaksberger (Flaksberger, 1935; Flaksberger et al., 1939). The system is based on taking into account the genomic composition of species and the presence or absence of a number of major genes that govern systematically important traits.

In accordance with this system of the genus, durum wheat (T. durum Desf.) is treated as a separate species in the rank of the species, which was first described by the French botanist R.L. Desfontaines (1798). The species includes two subspecies: subsp. durum and subsp. horanicum Vav. The latter is a subspecies of the most dense-ear wheats, with a complex of specific morphological characters. Subsp. durum is a subspecies of durum wheat proper, within which six groups of botanical varieties (convarieties) are distinguished, namely convar. durum, durocompactum Flaksb., aglossicon Dorof. et A. Filat., villosum (Jakubz.) Dorof. et A. Filat., falcatum (Jakubz.) Dorof. et A. Filat., caucasicum (Dorof.) Dorof. In turn, convar. durum includes three subconvarieties: subconvar. durum, muticum (Orlov) Dorof. et A. Filat., and duroramosum Dorof. (Table 1). At the time of the creation of the classification by V.F. Dorofeev et al. (1979), the genus T. durum Desf. numbered 120 botanical varieties and 29 forms in 20 varieties. As a result of subsequent studies, 11 more botanical varieties and 12 forms were identified (Lyapunova, 2017, 2019).

**Table 1.**
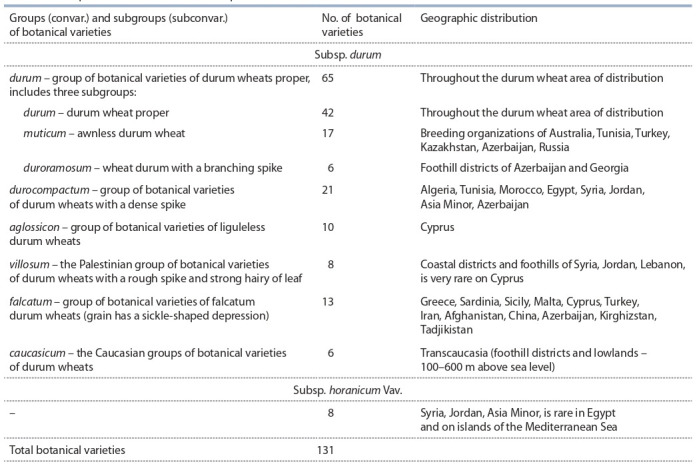
Intraspecific differentiation of the species Triticum durum Desf

The classification, which is most often referred to as the Classification of Triticum by Dorofeev et al., belongs to a number of the main modern classifications of the genus Triticum L. This was the first standardized classification that contained all known intraspecific taxa of wild and cultivated wheat species. A similar classification, a development of previous classifications based on the use of a comparative genetic approach, was proposed by N.P. Goncharov (Goncharov, 2002, 2005, 2009; Goncharov et al., 2007). In contrast to hexaploid wheats, the species classification of which can be constructed using only five main genes (Goncharov, 2011), in tetraploid species only Polish wheats and Ispahan emmer wheat can differ oligogenically (Watanabe et al., 1996; Watanabe, 1999). In all other species, only a part of taxonomically important traits has simple genetic control. This refers, e.g., to tetra-awnedness in the majority of T. carthlicum Nevski varieties (Haque et al., 2011)^1^ , purple grain of T. aethiopicum Jakubz. (Lachman et al., 2017), and ear branching in T. turgidum L. (Haque et al., 2012). At the same time, the botanical varieties identified by us have a simple control of characters. For example, ligulessness (Barulina, 1937; Watanabe et al., 2004) or awnlessness (Goncharov et al., 2003).

The gene has been recently introgressed into hexaploid wheat and mapped (Dobrovolskaya et al., 2020).


Such a detailed classification makes it possible to identify a wide diversity when working with the genus Triticum L. as a whole and/or with its individual species, which is especially important for large-scale collections preserved in genetic seed banks.

The use of intraspecific classification of the genus Triticum L. greatly simplifies the identification of the VIR collection accessions introduced from various sources, or when checking accession identity after regeneration in the field. However, apart from the researchers at the Department of Wheat Genetic Resources of VIR, few people use this approach in their practical work, and there are several reasons for this. First, both the monograph itself (Dorofeev et al., 1979) and the accompanying “Identifier of Wheat” (Dorofeev et al., 1980) have not been reprinted for more than 40 years and became a bibliographic rarity, which makes it difficult for national breeders and other wheat researchers to use it (Chikida, 2020). After the collapse of the USSR, the genetic banks of the COMECON (Council for Mutual Economic Assistance – an economic organization from 1949 to 1991 under the leadership of the Soviet Union that comprised the countries of the Eastern Bloc along with a number of socialist states elsewhere in the world) countries stopped working according to a common pattern, although many of them continue to use the system developed by V.F. Dorofeev et al. (1979). Second, there is still no translation of these works into English, although there was an international project on the translation of this monograph (Knüpffer et al., 2003), which makes it impossible for the staff of foreign genetic seed banks to get acquainted with this classification. Third, only the long-term practice of identifying accessions by the name of a botanical variety makes it possible to carry out this laborious work promptly and without difficulty. For instance, durum wheat alone requires remembering names of 131 varieties and their meaning. One of the ways to reduce the number of hard-toremember names may be unification as a standardization method aimed at reducing the number of objects by combining several characters. It assumes selection of the optimal number of objects, botanical varieties in our case, limited to a reasonable minimum and leads to a certain uniformity. This greatly simplifies the practical use of the classification. 

The objective of this work is to acquaint the reader with the intraspecific classification of durum wheat (Triticum durum Desf.) developed at VIR, and to offer its simplified analog based on the identification and illustration of the main and additional morphological characters of the ear and kernel.

## Materials and methods

Here, we propose a unified intraspecific classification of the durum wheat species, based on the description of only 16 main botanical varieties which have the most commonly occurring sets of morphological characters of the ear and kernel, and retain their author’s name (Table 2). The remaining botanical varieties, which have additional characters, get their name by the addition of this or that additional character to the main abbreviated Latin name (Table 3). Such a way of describing and quickly memorizing intraspecific diversity was proposed for common wheat in (Zuev et al., 2019). This work has been successfully published twice and is in great demand both domestically and among employees of foreign genetic seed banks.

**Table 2.**
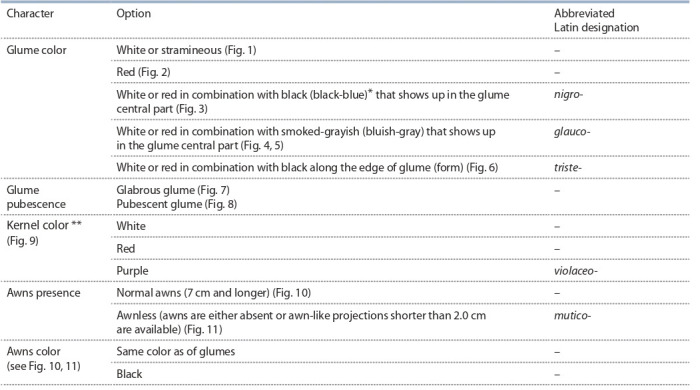
The main spike and kernel characters used to describe the intraspecific diversity of durum wheat * The blue tint is due to the presence of a waxy coating on the spike glumes ** Kernels with light yellow, yellow and amber-yellow color are attributed to the group of white-colored ones; while those with light brown, brown and amberbrown color are grouped as red-colored kernels (The International Comecon List of Descriptors…, 1984). Durum wheat kernels are mostly vitreous, therefore the color that is defined as white, is in fact amber-yellow.

**Table 3.**
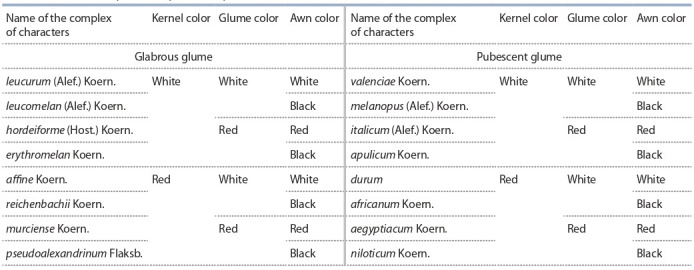
The most frequent complexes of spike and kernel characters in durum wheat and their Latin name

## Results

**Basic and additional morphological characters of durum wheat**

The intraspecific description system is based on botanical varieties, the names of which are determined by a set of morphological characters of the ear and kernel. These sets were distinguished by a combination of such features as the presence or absence of glume pubescence, glume color (white, red, smoky gray, or black on a white or red background), the presence or absence of awns on the lemma, their color (matching the color of the glume, or black), and the kernel shape and color (white, red or purple) (see Table 2).

A description of each botanical variety must include a set of main features: the presence/absence of glume pubescence, the color of the glume and kernel, the presence/absence of awns on the lemma, and the color of awns. The set of characters revealed by a specimen is designated by the corresponding Latin name given by the author (see Table 3).

To describe a specimen that possesses one of these sets of characters, but in combination with an additional character, like color of the glume, different length of awns, their color, etc., abbreviated Latin names of these characters are used (Table 4). In the case of durum wheat, these names are added to the name of the main set in the case of peduncle pubescence (piloso-) or awns smoothness (levi-), or when they determine the names of groups or subgroups of botanical varieties, i. e., dense-eared (-compactus); with the crescent-shaped kernel (falcato-); with the branching ear (ramoso-), non-ligulate (quasi-), with the densely pubescent leaf blade and sheath of the leaf, and with the hard glume (villoso-). Along with the characters of the ear and kernel, Table 4 contains that of the ligula absence, which is the only character of the leaf taken into account when describing botanical varieties.

**Table 4.**
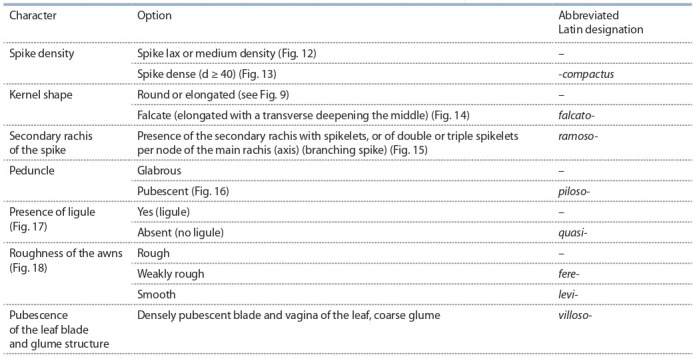
Additional spike and kernel characters used to describe the intraspecific diversity of durum wheat

**Fig. 1.**
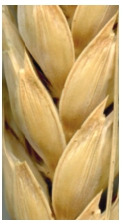
White glume color.

**Fig. 2.**
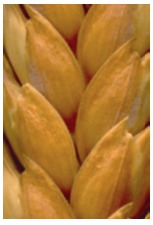
Red glume color.

**Fig. 3.**
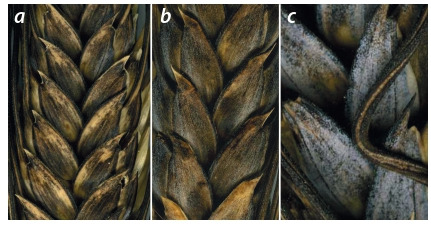
White (a) or red (b) color of glume in combination with black color or blue-black color (c).

**Fig. 4.**
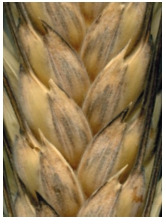
White glume color in combination with smoked-grayish color (glauco-).

**Fig. 5.**
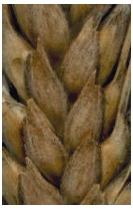
Red glume color in combination with smoked-grayish color (glauco-)

**Fig. 6.**
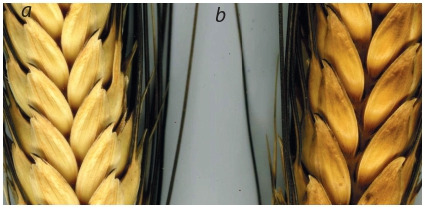
White (a) or red (b) glume color in combination with black along edge (triste-).

**Fig. 7.**
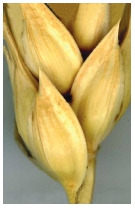
Glabrous glume.

**Fig. 8.**
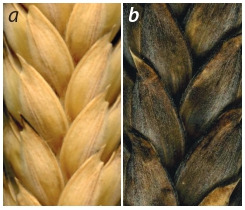
. Pubescent glume: a – white spike, b – black spike.

**Fig. 9.**
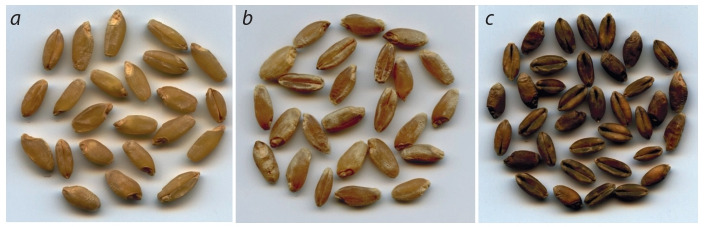
Color of durum wheat kernels: white (a), red (b), purple (в).

**Fig. 10.**
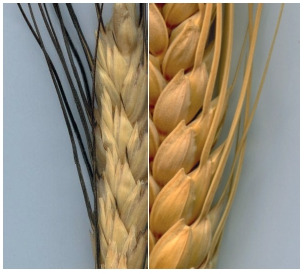
Awned durum wheat.

**Fig. 11.**
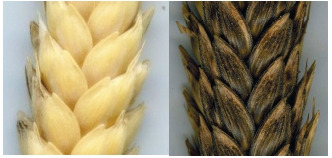
Awnless durum wheat.

**Fig. 12.**
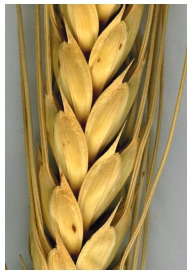
Lax spike of durum wheat.

**Fig. 13.**
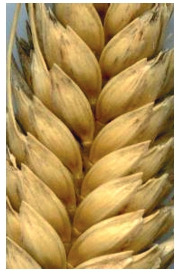
Dense spike of durum wheat.

**Fig. 14.**
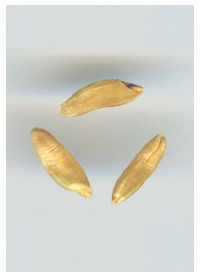
Falcate kernel shape.

**Fig. 15.**
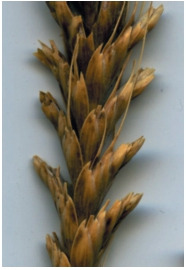
Branching spike of durum wheat.

**Fig. 16.**
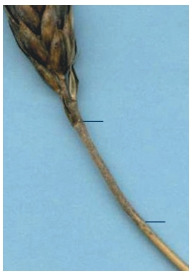
Pubescent peduncle.

**Fig. 17.**
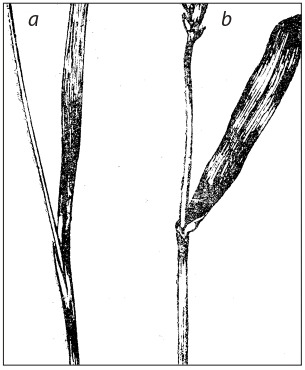
Non-ligulate (a) and ligulatee (b) plant of durum wheat (from: Flaksberger, 1935).

**Fig. 18.**
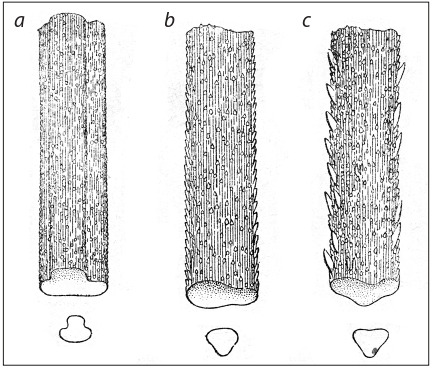
Awn: smooth (a), weakly rough (b), strongly rough (с) (from: Flaksberger, 1935).

**Unified intraspecific classification of durum wheat (Triticum durum Desf.)**

The proposed unified intraspecific classification is a simplified analog of the durum wheat key (Dorofeev et al., 1980). The whole diversity is arranged in the form of tables, where the names of varieties according to K.A. Flaksberger (1935) and V.F. Dorofeev et al. (1979) are given for comparison, which allows a user to establish a correspondence between the form being described and the botanical variety. The botanical varieties are presented in accordance with the main characters in the following sequence: the awned and awnless forms are presented in Table 5 and Supplementary^2^.

**Table 5.**
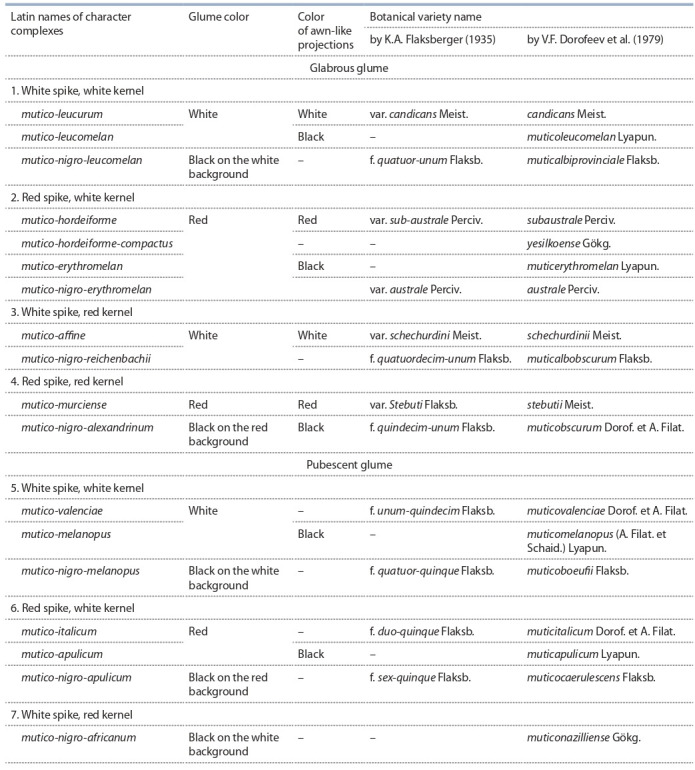
Character complexes found in the awnless durum wheat accessions

^2^ Supplementary material is available at: http://vavilov.elpub.ru/jour/manager/files/Suppl_Lyapunova_Engl.pdf


In the first place, these tables present botanical varieties with non-pubescent glumes and different color combinations of the glume and kernel, and then those with the pubescent glumes in the same order.

Glabrous glume

- White spike, white kernel.
- Red spike, white kernel.
- White spike, red kernel.
- Red spike, red kernel.
- White spike, purple kernel.
- Red spike, purple kernel.
- Pubescent glume: White spike, white kernel.
- Red spike, white kernel.
- White spike, red kernel.
- Red spike, red kernel.

All of the above main characters have simple genetic control (МсIntosh et al., 2020).

## Conclusion

Acquaintance with the durum wheat intraspecific classification, which was created at VIR and contained all the known intraspecific taxa of the time as well as the subsequently added ones, will make it possible to analyze all the intraspecific diversity of the main cultivated tetraploid species Triticum durum Desf. The proposed simplified analog version, based on the identification of the main and additional morphological characters of the ear and kernel, can help any user simplify the systematization of the intraspecific diversity contained in any collection and easily navigate it.

## Conflict of interest

The authors declare no conflict of interest.
